# Cardiomyopathy in offspring of diabetic rats is associated with activation of the MAPK and apoptotic pathways

**DOI:** 10.1186/1475-2840-8-43

**Published:** 2009-07-31

**Authors:** Benjamin E Reinking, Elesa W Wedemeyer, Robert M Weiss, Jeffrey L Segar, Thomas D Scholz

**Affiliations:** 1 Departments of Pediatrics and Internal Medicine, University of Iowa, Iowa City, IA USA 52242-1083, USA; 2 UIHC, Division of Pediatric Cardiology, 200 Hawkins Drive, 2801 JPP, Iowa City, IA 52242-1083, USA

## Abstract

**Background:**

Maternal diabetes affects the developing fetal cardiovascular system. Newborn offspring of diabetic mothers can have a transient cardiomyopathy. We hypothesized that cardiomyopathic remodeling is associated with activation of the mitogen activated protein kinase (MAPK) signaling and apoptotic pathways.

**Methods:**

To evaluate the effects of moderate and severe maternal hyperglycemia, pregnant rats were made diabetic with an injection of 50 mg/kg of streptozotocin. Moderately well controlled maternal diabetes was achieved with twice daily glucose checks and insulin injections. No insulin was given to severely diabetic dams. Offspring of moderate and severe diabetic mothers (OMDM and MSDM, respectively) were studied on postnatal days 1 (NB1) and 21 (NB21). Echocardiograms were performed to evaluate left ventricular (LV) dimensions and function. Myocardial MAPK and apoptotic protein levels were measured by Western blot.

**Results:**

OMDM had increased cardiac mass at NB1 compared to controls that normalized at NB21. OSDM demonstrated microsomia with relative sparing of cardiac mass and a dilated cardiomyopathy at NB1. In both models, there was a persistent increase in the HW:BW and significant activation of MAPK and apoptotic pathways at NB21.

**Conclusion:**

The degree of maternal hyperglycemia determines the type of cardiomyopathy seen in the offspring, while resolution of both the hypertrophic and dilated cardiomyopathies is associated with activation of MAPK signaling and apoptotic pathways.

## Background

Infants of diabetic mothers (IDM) represent a high-risk group of patients with an increased incidence of perinatal morbidity and mortality [1]. Even with good maternal serum glucose control, the offspring are at increased risk for complex multisystem medical problems including macrosomia, hypoglycemia, hypocalcaemia, and respiratory distress syndrome [2]. Although not commonly seen in current medical practice, very poorly controlled gestational diabetes results in small for gestational age infants with relative sparing of internal organs such as the heart and significant multisystem medical problems [3].

The cardiomyopathy seen in very poorly controlled gestational diabetes is likely different from the hypertrophic cardiomyopathy (HCM) found in better controlled IDM [2,4-6]. The HCM seen in infants of well controlled diabetic mothers is characterized by asymmetric septal hypertrophy with individual myocyte hypertrophy and limited myofiber disarray [6]. The functional significance of the HCM is variable, ranging from a clinically insignificant echocardiographic finding to congestive heart failure as a result of left ventricular outflow tract obstruction and diastolic dysfunction [7]. Regardless of severity, the cardiac hypertrophy is transient, with echocardiographic resolution by six months of age [6]. While the morphologic characteristics of the cardiomyopathy seen in IDM are well described, the molecular mechanisms regulating its development and resolution are currently unknown.

The heart is capable of remodeling in response to physiologic and pathologic stimulation. The mitogen activated protein kinase (MAPK) pathways play a key role in the response of cardiac myocytes to these stressful stimuli [8,9]. The most studied terminal kinases of these pathways: extracellular signal regulated protein kinases (ERK), c-Jun NH_2_-terminal kinases (JNK), and p38, activate transcription factors that ultimately affect gene expression [10]. In general, ERK activation (or phosphorylation) is pro-growth and has been implicated in hyperplasia of immature cardiomyocytes and hypertrophy of terminally differentiated cardiomyocytes [11]. The roles of JNK and p38 are less clear. *In vitro*, JNK and p38 activation promotes hypertrophy although *in vivo *these pathways appear to prevent hypertrophy [12]. In addition to the roles these pathways play in cell growth and proliferation, the MAPK signaling pathways are powerful regulators of apoptosis [13]. While apoptosis has been implicated in the development of some forms of cardiomyopathy, its role in the development and resolution of the HCM seen in IDM is unknown [14,15].

In order to explore the role of the MAPK and apoptotic pathways in the development and resolution of the cardiomyopathy seen in infants of diabetic mothers, we developed two rat models of gestational hyperglycemia – one with very poorly controlled glucose with maternal ketosis and a second model with moderately well controlled glucose levels and no maternal ketosis. We hypothesized that the offspring of both the moderately and severely diabetic mothers would demonstrate a cardiomyopathy whose postnatal resolution was associated with activation of the MAPK signaling and apoptotic pathways.

## Methods

### Animal Preparation

To develop a moderate level of maternal hyperglycemia (serum glucose in the 200 to 400 mg/dl range with no maternal ketosis), timed, pregnant Sprague-Dawley rats obtained from Charles River (Charles River Laboratories, Wilmington, MA) were injected with 50 mg/kg streptozotocin (STZ) intraperitoneally on day 12 of gestation. Streptozotocin (Sigma Chemical, St. Louis, MO) was dissolved in citrate buffer (pH 4.5) and injected within 5 minutes to prevent its degradation. Serum glucose levels were monitored twice daily by sampling from tail-nicking (OneTouch Ultra, LifeScan, Miltitas, CA) and insulin given twice daily based on a sliding scale. Regular Humulin (Eli Lilly and Company, Indianapolis, IN) was given for the morning dose (1 – 3 units subcutaneously) and long-acting Lantus insulin (Aventis Pharmaceuticals, Inc.) was given in the early evening (1 – 3 units subcutaneously). Typically, decreased insulin dosing was needed in the one to two days prior to delivery. Control pregnant rats were given the identical volume of citrate buffer without STZ, had serum glucose monitored twice daily, and given twice daily saline injections. Offspring were studied at postnatal day 1 (NB1) and postnatal day 21 (NB21). On the first day of life, all pups to be studied at NB21 were transferred to foster dams obtained at the same time as the STZ and sham injected animals whose newborns were removed. At NB1 and NB21, a subset rat pups were weighed and then euthanized in a carbon dioxide chamber followed by decapitation for morphometric measurements including body and whole heart weight (Control NB1: N = 102; OMDM NB1: N = 181; Control NB21: N = 28; OMDM NB21: N = 24).

For the model of severe maternal hyperglycemia with ketosis, intravenous injections of streptozotocin (50 mg/kg) or normal saline were given to pregnant rats by Zivic-Miller Laboratory (Pittsburgh, PA) employees on day 7 of gestation. Maternal glucose levels were measured on day 15 of gestation and on the day following parturition as above. The upper limit of the glucometer is 600 mg/dl. Any reading greater than this value was reported as "high" by the meter. Any streptozotocin-injected rats that were not diabetic (greater than 400 mg/dl on day 15 of gestation) were excluded from the study. Blood was obtained from a subset of maternal rats to test for the presence of ketones (Acetest; Bayer Corp, Pillchant IN). Offspring at NB1 and NB21 were handled as described above. Morphometric measurements were made on a subset of animals (Control NB1: N = 72; OSDM NB1: N = 37; Control NB21: N = 35; OSDM NB21: N = 15). Additional hearts from both models of maternal hyperglycemia were also excised and frozen for Western blot analysis.

All procedures were performed within the regulations of the Animal Welfare Act and the National Institutes of Health Guide for the Care and Use of Laboratory Animals and were approved by the University of Iowa Animal Care and Use Committee.

### Echocardiograms

Echocardiography was performed using a Philips Sonos 5500 imager fitted with a 15 MHz linear array probe on OSDM NB1 (n = 13 control, 6 experimental) and NB21 (n = 3 control, 6 experimental) rat pups [16,17]. When OMDM animals were studied, a new imaging system with better spatial and temporal resolution was available. This imaging was performed with a Vevo 770 high resolution ultrasound imaging system (VisualSonics, Inc., Toronto, Ontario) fitted with a 24 MHz mechanical transducer (NB1: n = 7 control, 5 experimental; NB21: n = 7 control, 7 experimental). Short-axis images were acquired parallel to the mitral valve plane so as to obtain the largest cross-sectional image of the left ventricle that did not contain the mitral valve. Long-axis views were obtained perpendicular to the mitral valve plane and were deemed optimal when the diastolic apex-to-base length was longest and when both mitral and aortic valves were visible. Ejection fraction (EF) and left ventricular mass (LVM) were determined using Simpson's rule from 2-D images obtained on the Sonos 5500 and from m-mode images on the Vevo 770 using analysis packages available on each system.

### Western blots

Immunoblots were prepared as described previously [18]. Briefly, myocardial samples were homogenized in the presence of protease inhibitors including soybean trypsin inhibitor, leupeptin, and PMSF in 50 mM Tris/10 mM EDTA/150 mM NaCl/0.1% mercaptoethanol and then sonicated for 20 seconds. Following centrifugation, total protein of the supernatant was quantitated spectrophotometrically. 20 mcg of protein were separated by SDS-PAGE and transferred to a nitrocellulose membrane. Membranes were blocked with Odyssey Blocking Buffer (Part No. 927–40000, Li-Cor Biosciences, Lincoln, NE) for one hour at room temperature and then incubated in primary antibody overnight at 5°C. Bound primary antibody was detected by incubation with infrared-labeled secondary antibodies (IRDye 800 or IRDye 700 700DX, Li-Cor Biotechnology, Lincoln, NE), read, and quantitated with a Li-Cor Odyssey Imaging System (Li-Cor Biosciences, Lincoln, NE).

Primary antibodies that were utilized included antibodies specific to total and phosphorylated terminal MAP kinases (ERK1/2, JNK 1/2, and p38), and caspases 8 and 3. [Antibodies to ERK 1/2 (sc492), pERK (sc7383), JNK1/2 (sc571), and pJNK (sc625) were obtained from Santa Cruz Biotechnologies, Inc., Santa Cruz CA; antibodies to active caspase 3 (55–9565) and active caspase 8 (51–8125) were obtained from BD Biosciences Pharmingen, San Jose CA]. All primary antibodies were used at a 1:1000 dilution.

### Statistics

All data are displayed as mean ± standard error of the mean (S.E.M.). Comparisons between the OMDM and OSDM and their controls were made using an unpaired t-test. Significant differences were identified at the p < 0.05 levels

## Results

### Maternal data

Blood glucose was measured twice daily between day 12 of pregnancy and delivery to monitor the effectiveness of insulin therapy in the moderately diabetic model. Generally, blood glucose values were in the 200–400 mg/dl range, although higher and lower values occurred. Periodic screening for ketones in the pregnant dams was negative.

In the severely diabetic model, maternal blood glucose levels were measured to document hyperglycemia on day 15 of gestation and postnatal day 1. Blood glucose ranged from 83 – 104 mg/dl in control rats and 458 - >600 mg/dl in diabetic dams at both time points, with the majority of the values >600 mg/dl. Blood from a subset of maternal rats obtained on the day of delivery (n = 3 control, 3 diabetic) was positive for moderate ketones in all diabetic samples and negative in control samples. In addition, maternal severely diabetic rats generally appeared cachectic, although morphometric measurements on the mothers were not made.

### Morphometric data

Maternal diabetes affected the body weight and heart weight of the rat pups (Table 1). The OMDM had increased heart weight and heart weight to body weight ratio (HW:BW) at NB1 when compared to control animals. At NB21, there continued to be relative organomegally with an increased HW:BW in the OMDM. There was not a significant difference in the body weight of OMDM and control animals at either time point.

**Table 1:** Morphometric Data from offspring of control and diabetic mothers.

	Offspring of **Moderately** Diabetic Mothers			Offspring of **Severely** Diabetic Mothers		
	Body Weight (g)	Heart Weight (mg)	HW/BW (mg/g)	Body Weight (g)	Heart Weight (mg)	HW/BW (mg/g)

NB1 Control	7.0 ± 0.1	43 ± 1	6.1 ± 0.1	8.3 ± 0.1	49 ± 1	5.8 ± 0.1

NB1 ODM	7.1 ± 0.1	48 ± 1*	6.7 ± 0.1*	5.9 ± 0.2*	39 ± 2*	6.6 ± 0.2*

NB21 Control	58.6 ± 1.1	294 ± 7	5.0 ± 0.1	43.5 ± 3.2	214 ± 11	5.0 ± 0.1

NB21 ODM	55.7 ± 1.1	309 ± 6	5.6 ± 0.1*	41.9 ± 6.2	230 ± 16	5.6 ± 0.4*

All values are mean ± SE; NB1 = 1 day old animals, NB21 = 21 day old animals, HW/BW – heart weight to body weight ratio, ODM – offspring of diabetic mothers. *p < 0.05 versus control by unpaired t-test.

Conversely, the OSDM had decreased body weight and decreased heart weight when compared to control animals at NB1. There was relative sparing of myocardial mass when compared to body weight resulting in an increased HW:BW at this time point. Despite normalization of the absolute heart weight and body weight by NB21, the increase in the HW:BW persisted in the OSDM when compared to controls.

### Echo data

Maternal diabetes also affected the left ventricular size and function of the rat pups, as determined by echocardiography. The OMDM and control animals had similar left ventricular size and ejection fraction at both the NB1 and NB21 time points (Table 2). The newborn OSDM, however, had a significantly decreased left ventricular ejection fraction compared to controls. This was accompanied by a significant increase in left ventricular end systolic volume in the OSDM (control: 2.0 ± 0.3 mcl; OSDM: 4.3 ± 1.0 mcl; p < 0.05) suggesting that the OSDM developed a dilated cardiomyopathy. By 21 days of age, there was echocardiographic resolution of the cardiomyopathy in the OSDM, with normalization of the ejection fraction (Table 2) and end systolic volume (control: 33 ± 12 mcl; OSDM: 24 ± 8 mcl; p = 0.6).

**Table 2:** Echocardiographic data from offspring of control and diabetic mothers.

	EF – Moderately Diabetic Model (%)	EF – Severely Diabetic Model (%)	LVM – Moderately Diabetic Model (mg)	LVM – Severely Diabetic Model (mg)
NB1 Control	73 ± 4	88 ± 1	38 ± 4	31 ± 2

NB1 ODM	69 ± 3	76 ± 4*	8 ± 3	33 ± 5

NB21 Control	68 ± 4	80 ± 4	197 ± 13	192 ± 7

NB21 ODM	70 ± 2	86 ± 5	220 ± 16	192 ± 12

All values are mean ± SE; NB1 – 1 day old animals, NB21 – 21 day old animals, EF = ejection fraction, LVM = left ventricular mass. *p < 0.05 versus control by unpaired t-test

No significant differences were seen in echocardiographic-calculated left ventricular mass between controls and ODM at both ages in either the severely or moderately diabetic groups (Table 2). Of note, the m-mode method of calculating ejection fraction that was used in the OMDM gave consistently lower values compared to the modified Simpson's method from 2-D images used in the OSDM animals.

### MAPK Pathways

**Figure 1:**
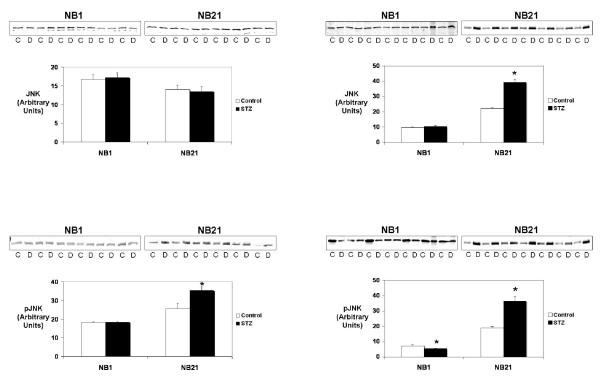
**Total and active c-Jun NH2-terminal kinase (JNK, and pJNK, respectively) protein levels were measured by immunoblot in hearts from control (C) and offspring of either moderately or severely diabetic dams (D) at 1 and 21 days of life (NB1 and NB21, respectively)**. The immunoblots are shown at the top with the bar graphs indicating protein levels expressed in arbitrary units. Molecular weights: JNK – 48 kD, pJNK – 46 kD. *p < 0.05 versus control value by unpaired t-test.

**Figure 2:**
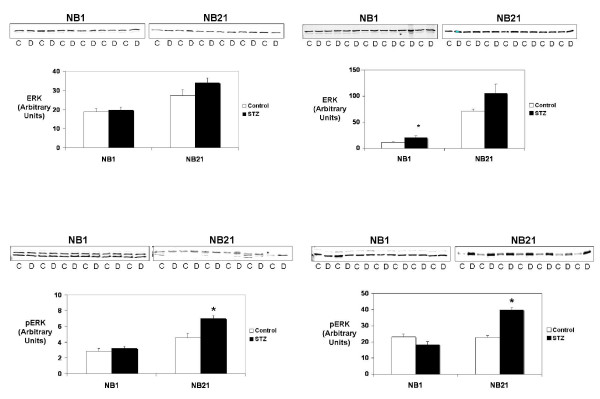
**Total and active extracellular signal-regulated kinase (ERK, and pERK, respectively) protein levels were measured by immunoblot in hearts from control (C) and offspring of either moderately or severely diabetic dams (D) at 1 and 21 days of life (NB1 and NB21, respectively)**. In NB1 myocardial samples, ERK1 and ERK2 were both seen and quantitated as a single band. The immunoblots are shown at the top with the bar graphs indicating protein levels expressed in arbitrary units. Molecular weights: ERK1 – 42 kD, ERK2 – 44 kD, pERK – 43 kD. *p < 0.05 versus control value by unpaired t-test.

Maternal diabetes altered MAPK protein activation in the newborn OMDM and OSDM with evidence of continued ventricular remodeling at NB21. Steady state levels of the total and activated MAPK proteins were measured using Western blot analysis. When present, ERK isomers were analyzed as a single band. A similar pattern of MAPK protein activation was seen in the offspring diabetic mothers, regardless of severity (Figures 1 and 2). In both OMDM and OSDM, animals at 21 days of age were found to have significantly increased activation of both JNK and ERK. No change was found in pJNK or pERK levels in NB1 animals from either model except a small but significant decrease in pJNK in the NB1 OSDM (Figure 1).

Total MAPK protein levels remained fairly stable with significant differences between offspring of diabetic mothers and control animals only found for JNK in NB21 and ERK in NB1 of OSDM.

Although not shown, low levels of p38 and phosphorylated p38 were found in all animals studied although no differences were seen between offspring of diabetic mothers and control animals from either model.

### Apoptosis protein levels

**Figure 3:**
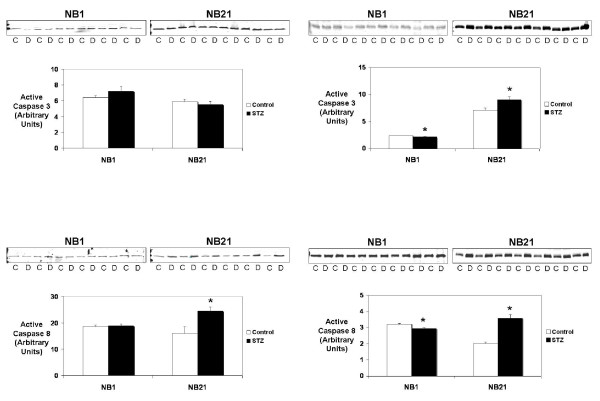
**Active Caspase 3 and active Caspase 8 protein levels were measured by immunoblot in hearts from control (C) and offspring of either moderately or severely diabetic dams (D) at 1 and 21 days of life (NB1 and NB21, respectively)**. The immunoblots are shown at the top with the bar graphs indicating protein levels expressed in arbitrary units. Molecular weights: caspase 3 – 35 kD, caspase 8 – 55 kD. *p < 0.01 versus control value by unpaired t-test.

The levels of active, or cleaved, caspase 3 and caspase 8 were determined for animals from both models and at both time points. In NB1 pups, levels of all measured proteins were similar between groups, although small but significant decreases in caspase 3 and 8 were seen in the NB1 OSDM (Figure 3). By 21 days of age, however, apoptotic pathways were found to be significantly activated (Figure 3). In the OMDM, active caspase 8 levels were significantly elevated at 21 days of age. Both active caspase 3 and active caspase 8 protein levels were significantly elevated in the NB21 pups that were OSDM.

## Discussion

Maternal diabetes creates an adverse environment for fetal development resulting in a number of anatomic, hematologic, and physiologic abnormalities in the newborn [19]. The effects of uncontrolled or long-standing diabetes on the fetus and the neonate are quite different from those found in well-controlled maternal diabetes. Prior to the use of insulin, few diabetic women conceived and even fewer carried a pregnancy to term [19]. In performing a post mortem quantitative morphologic study on infants of diabetic mothers and comparing them to controls matched for gestational and postnatal age, Naeye identified two populations of infants of diabetic mothers [3]. The first group of infants had a body weight that was 141% of the control values. In addition, these infants had organomegally with heart weights that were 174% of the control values. This group likely represented mothers with residual insulin function whose serum glucose values were elevated but did not become ketotic. The second group consisted of underweight infants with body weights 61% of control values and heart weights that were 51% of controls. This group was likely more severely ill and was thought to have placental insufficiency due to their degree of illness. The decrease in cardiac mass in the newborns was attributed to myocyte death [3].

The findings of our study are consistent with Naeye's observations. Specifically, OSDM had a decrease in body weight and heart weight when compared to control pups. Due to a relative sparing of heart weight compared to body weight, however, there was a significant increase in the heart weight to body weight ratio at both time points. These findings were associated with decreased left ventricular systolic function echocardiographically in the immediate newborn period. The difference between control and OSDM heart weight-to-body weight and cardiac function is likely related to the poorly controlled maternal diabetes and associated maternal anorexia, undernutrition and/or placental insufficiency. This theory is supported by both human and animal data. Echocardiographic studies have found a relative sparing of cardiac weight when compared to body weight in human pregnancies complicated by poorly controlled maternal diabetes and intrauterine growth restriction (IUGR) [20]. IUGR alone is also associated with decreased cardiac function that worsens with advancing gestation [21].

Several studies have described the cardiomyopathy seen in the macrosomic offspring of mothers with moderately well controlled gestational diabetes [2,4-6,22]. The HCM in these infants is characterized by asymmetric hypertrophy of the ventricular septum [6]. While increased septal thickness was not found by echo in the OMDM in this study, an increase in cardiac mass was seen at one-day of age in the OMDM compared to controls. As in humans, variability in septal thickness between animals may have prevented the identification of significant differences in this measurement between groups.

While multiple studies have described the morphologic and histologic characteristics of the cardiomyopathy seen in infants of diabetic mothers, no study to our knowledge has described the molecular mechanisms that correlate with the cardiomyopathic changes. The striking finding of this study is that regardless of the extent of intrauterine insult to the heart due to maternal hyperglycemia, the postnatal remodeling process was accompanied by activation of the MAPK signaling pathways. The MAPK signaling pathways have multiple effects on cardiac myocyte growth, proliferation and apoptosis and have been implicated in other forms of cardiomyopathy [14,15]. We found that at one day of age, the hypertrophic cardiomyopathy in OMDM and the dilated cardiomyopathy in OSDM was associated with limited changes in MAPK activation, although small changes were found at NB1 in OSDM in total ERK and active (phosphorylated) JNK (Figures 1 and 2). In both models, the resolution of the cardiomyopathy at NB21 was associated with significant up regulation of both active JNK (pJNK) and active ERK (pERK). Levels of active P38 were unchanged at both time points. The downstream targets of these signaling proteins that contribute to the cardiac remodeling in the offspring of diabetic dams are currently not known.

ERK has the most well defined role of the MAPK proteins. In general, ERK is considered to be progrowth, promoting hyperplasia in uninucleated myocytes and hypertrophy in binucleated myocytes [11,23]. Bueno *et. al*. examined the effects of ERK activation in transgenic mice with cardiac specific expression of the ERK activator, MEK1. In addition, they activated ERK in cultured myocytes by transfecting with MEK1 adenovirus. Both experiments demonstrated that the MEK1/ERK signaling pathway stimulated physiologic hypertrophy associated with augmented cardiac function and partial resistance to apoptosis [24]. In addition, ERK was found to play a key role in IGF-1 induced cardiac myocyte proliferation [25]. The significant increase in pERK in NB21 offspring in both models may reflect a general decrease in myocyte cell number *in utero *due to maternal hyperglycemia that is compensated for by an increase in pERK-driven myocyte proliferation postnatally. Measurement of myocyte size in the OMDM would help to define whether individual myocyte hypertrophy is present in these animals, which would be necessary if cardiac hypertrophy, as seem in the NB1 pups, was accompanied by decreased myocyte number.

The roles of JNK and p38 are less well defined than that of ERK. As recently reviewed by Liang and Molkentin, p38 and JNK have differing roles *in vivo *and *in vitro *[26]. *In vivo*, activation of p38 and JNK appears to inhibit hypertrophy and promote apoptosis, while *in vitro*, activation of these pathways appears to promote hypertrophy [8,12,27,28]. Although phosphorylated p38 protein levels were unchanged, there was a striking increase in the levels of active JNK (pJNK) at NB21 in both models. Thus, at 21 days of age there appears to be activation of competing regulatory proteins in the hearts of the offspring of diabetic mothers, regardless of the degree of maternal hyperglycemia, with ERK being prohypertrophic and antiapoptotic and JNK being antihypertrophic and proapoptotic. Whether these competing influences work to appropriately couple myocyte hyperplasia with myocyte apoptosis, as has been suggested occurs during normal cardiac development [29], is not known.

We found indications that activation of apoptotic pathways was important to the cardiac remodeling seen in both OMDM and OSDM (Figure 3). Up-regulation of apoptosis was assessed by measuring protein levels of the initiator caspase 8 and the effector caspase 3. In the OMDM, a significant increase was found in active caspase 8 while both active caspase 3 and 8 were significantly increased in OSDM in NB21 hearts. This suggests that despite activation of competing MAP kinase signaling pathways in the NB21 offspring of diabetic mothers, the JNK signaling to promote apoptosis was more robust than ERK signaling.

### Limitations

The results of this study may have been affected by the overall health of the diabetic dams, particularly in the severely hyperglycemic model where they were likely malnourished due to illness-induced anorexia. These issues are, however, clinically relevant and are seen in human pregnancies complicated by poorly controlled diabetes. Direct comparison of the consequences on the two models on the offspring is impacted by the different duration of hyperglycemia. The severely diabetics animals received streptozotocin on day 7 of pregnancy while the animals in the moderately diabetic model were made diabetic on day 12. It is interesting that similar changes in MAPK activation were found in the offspring suggesting that, despite the differing exposures to hyperglycemia, activation of the MAPK pathways may be common response in postnatal cardiac remodeling. However, limited time points were evaluated. Other important changes in the levels of inactive and active MAPK and apoptotic proteins could have occurred at time points not represented by our measurements.

## Conclusion

The degree of maternal hyperglycemia during pregnancy greatly impacts the type of cardiomyopathy found in the newborn. Moderately well controlled hyperglycemia, as occurs with most human pregnancies, results in hypertrophy of the neonatal heart. When the hyperglycemia is poorly controlled and impacts maternal, and likely fetal, nutrition, a dilated cardiomyopathy results. Both the cardiomyopathies are transient, and in the rat, have largely resolved by three weeks of age. The similar activation of the MAPK signaling and apoptotic pathways in these two models suggests that these pathways may have a wide-ranging impact on the ability of the heart to remodel. Exploration of MAP kinase and apoptotic activity in the remodeling process that occurs with other cardiomyopathies may help to understand how the heart responds to other insults that impact cardiac structure and function.

## Abbreviations

OMDM: offspring of moderately diabetic mothers; OSDM: offspring of severely diabetic mothers; APK: mitogen activated protein kinase; HW/BW: heart to body weight ratio; NB1: newborn day of life 1; NB21: newborn day of life 21; IDM: infants of diabetic mothers; HCM: hypertrophic cardiomyopathy; ERK: extracellular signal regulated protein kinases; JNK: c-Jun NH_2_-terminal kinases; HR: heart rate; EDV: end diastolic volume; ESV: end systolic volume; EF: ejection fraction; pERK: phosphorylated (or activated) ERK; pJNK: phosphorylated (or activated) JNK; IUGR: intrauterine growth restriction; STZ: streptozotocin.

## Competing interests

The authors declare that they have no competing interests.

## Authors' contributions

BR was involved in the animal preparation, echocardiographic experiments, data analysis, and data tabulation for the manuscript. BR also helped with manuscript preparation and editing. EW performed the majority of the immunoblots and helped with data analysis and figure preparation for the manuscript. RW performed most of the echocardiograms and helped to edit the manuscript. JS helped to conceive the studies, assisted with data analysis and manuscript preparation. TS also helped to conceive the studies, helped with data analysis and manuscript preparation and editing.

## Authors' information

BR, JS, and TS are all faculty in the Department of Pediatrics at the University of Iowa and members of the Program on Developmental Origins of Disease (PDOD). Among the interests of the PDOD are the short- and long-term consequences of being exposed to the adverse intrauterine environment caused by gestational diabetes mellitus.
